# Highly Sensitive Electrochemical Endotoxin Sensor Based on Redox Cycling Using an Interdigitated Array Electrode Device

**DOI:** 10.3390/mi14020327

**Published:** 2023-01-27

**Authors:** Kentaro Ito, Kumi Y. Inoue, Takahiro Ito-Sasaki, Miho Ikegawa, Shinichiro Takano, Kosuke Ino, Hitoshi Shiku

**Affiliations:** 1 Department of Frontier Science for Advanced Environment, Graduate School of Environmental Studies, Tohoku University, 6-6-11-604 Aramaki Aoba, Sendai 980-8579, Japan; 2 Center for Basic Education, Faculty of Engineering, University of Yamanashi, 4-3-11 Takeda, Kofu 400-8511, Japan; 3 Department of Biomedical Engineering for Health and Welfare, Graduate School of Biomedical Engineering, Tohoku University, 6-6-11-604 Aramaki Aoba, Sendai 980-8579, Japan; 4 Department of Biomolecular Engineering, Graduate School of Engineering, Tohoku University, 6-6-11-604 Aramaki Aoba, Sendai 980-8579, Japan

**Keywords:** endotoxin sensor, electrochemistry, *Limulus* amebocyte lysate reaction, redox cycling, interdigitated array electrode

## Abstract

The *Limulus* amebocyte lysate (LAL) reaction-based assay, the most commonly used endotoxin detection method, requires a skilled technician. In this study, to develop an easy-to-use and highly sensitive endotoxin sensor, we created an electrochemical endotoxin sensor by using an interdigitated array electrode (IDAE) device with advantages of amplifiable signals via redox cycling and portability. We added Boc-Leu-Gly-Arg-*p*-aminophenol (LGR-pAP) as an electrochemical substrate for an LAL reaction and detected *p*-aminophenol (pAP) released from LGR-pAP as a product of an endotoxin-induced LAL reaction via an IDAE device. The IDAE device showed a great redox cycling efficiency of 79.8%, and a 4.79-fold signal amplification rate. Then, we confirmed that pAP was detectable in the presence of LGR-pAP through chronoamperometry with the potential of the anode stepped from −0.3 to 0.5 V vs. Ag/AgCl while the cathode was biased at −0.3 V vs. Ag/AgCl. Then, we performed an endotoxin assay by using the IDAE device. Our endotoxin sensor detected as low as 0.7 and 1.0 endotoxin unit/L after the LAL reaction for 1 h and 45 min, respectively, and these data were within the cut-off value for ultrapure dialysis fluid. Therefore, our highly sensitive endotoxin sensor is useful for ensuring medical safety.

## 1. Introduction

An endotoxin is a component of the outer membrane of Gram-negative bacteria [1]. Endotoxin contamination of human blood triggers an inflammatory response called endotoxin shock [2,3,4]. Therefore, a highly sensitive method for detecting endotoxins in medical devices and drugs should be developed to ensure medical safety [5,6]. Currently, a *Limulus* amebocyte lysate (LAL) assay based on an endotoxin-induced enzymatic LAL cascade reaction is the most widely used endotoxin detection method [7]. In this assay, the endotoxin activates factor C, which in turn activates factor B. After the activation of a proclotting enzyme to a clotting enzyme by the activated factor B, the clotting enzyme reacts with coagulogen to produce coagulin gel. Endotoxins are detected by confirming gel formation with 180° standing inversion [8] or by measuring gel turbidity [9]. Additionally, a chromogenic endotoxin assay involving the LAL reaction is used by measuring the absorbance of *p*-nitroaniline released from Boc-Leu-Gly-Arg-*p*-nitroaniline (LGR-pNA) as a chromogenic substrate of the clotting enzyme [10,11]. However, these methods require a skilled technician, causing difficulties in detecting endotoxin contamination on-site.

To overcome this problem, we have developed an easy-to-use LAL-based endotoxin sensor by using an electrochemical chip device. A sensor using an electrochemical chip device enables user-friendly detection because it is small, portable, and convenient [12]. Previously, Li et al. electrochemically detected endotoxins as low as 6.1 × 10^−4^ ng/mL using a metal–organic framework [13]. Tian et al. developed an electrochemical aptasensor that could detect endotoxins as low as 5 fg/mL [14]. Xie et al. detected endotoxins on an electrochemical chip device with rolling circle amplification that had a detection limit of 4.8 fg/mL [15]. In our previous study, Boc-Leu-Gly-Arg-*p*-aminophenol (LGR-pAP) was used as a substrate of the clotting enzyme, and an endotoxin assay was performed by amperometrically detecting *p*-aminophenol released from LGR-pAP on an electrochemical chip device after 1 h of the LAL reaction [16]. Nonetheless, this method detected 10 endotoxin units (EU)/L, which was insufficient to reach the cut-off value of ultrapure dialysis fluid (1.0 EU/L), the highest-quality dialysis fluid according to the Committee of the Scientific Academy of the Japanese Society for Dialysis Therapy [17]. To improve LAL-based electrochemical sensors, we used the signal amplification system of substitutional stripping voltammetry. Substitutional stripping voltammetry amplifies the signal from pAP released from LGR-pAP by accumulating pAP signals as silver deposits. Consequently, endotoxin could be detected at 0.5 EU/L with 1 h of LAL reaction [18]. Furthermore, we developed an LAL-based electrochemical sensor by using the signal amplification of redox cycling in a nanoscale gap [19]. Redox cycling amplified the signal from pAP by repeating the redox reaction of pAP between two electrodes facing each other across a 190 nm gap; thus, 0.5 EU/L endotoxin could be detected within 30 min of the LAL reaction time. These electrochemical endotoxin sensors have achieved high sensitivity covering 1.0 EU/L; however, the structures of these devices are complicated. Especially, the device for redox cycling in a nanoscale gap required careful handling because of its fragile structure. Therefore, a simple electrochemical chip device with a signal amplification system for pAP detection is required to develop easy-to-use and highly sensitive endotoxin sensors.

**Figure 1:**
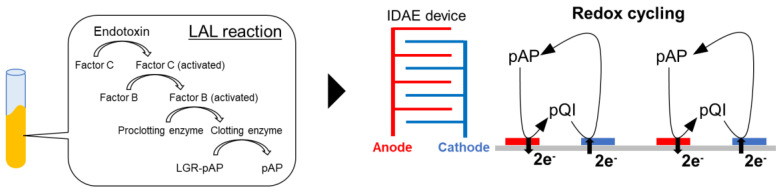
Schematic of the electrochemical endotoxin sensor using redox cycling with the IDAE device.

In this study, an LAL-based electrochemical endotoxin sensor with the signal amplification of redox cycling was developed using an interdigitated array electrode (IDAE) device. The IDAE device comprises two comb-shaped electrodes interdigitated alternately. Oxidized species at the anode of the comb-shaped electrode are reduced at the cathode of the comb-shaped electrode; thereafter, the reduced species are oxidized at the anode comb again. This cycling of redox reaction amplifies the electrochemical signal [20,21]. The IDAE device has the advantages of simplicity and robustness compared the device for redox cycling in a nanoscale gap used in our previous study [19,22,23,24]. This difference is attributed to the fragile nanometer-sized spatial structure of the device for redox cycling in a nanoscale gap, which is not present in the IDAE device. Then, an endotoxin assay was performed by detecting pAP released from LGR-pAP after the endotoxin-induced LAL reaction. First, the IDAE device was fabricated and characterized. Next, pAP was detected in the model solution of the LAL-reaction mixture composed of pAP and LGR-pAP. Finally, LAL-based endotoxin detection was performed via redox cycling by using the IDAE device. After the LAL reaction with LGR-pAP as the substrate of the clotting enzyme for 30 min, 45 min, and 1 h, pAP released from LGR-pAP was detected through redox cycling by the IDAE device that oxidized pAP at the anode and reduced *p*-quinoneimine (pQI) at the cathode (Figure 1).

## 2. Materials and Methods

### 2.1. Materials

The following materials were used in this study: United States Pharmacopeia reference standard endotoxin (USP-RSE) and a LAL-based endotoxin assay kit Endospecy-24S set comprising assay buffer and LAL reagents (Seikagaku, Tokyo, Japan); an endotoxin-free water (Otsuka Pharmaceutical, Tokyo, Japan); pAP and KCl (FUJIFILM Wako Pure Chemical, Osaka, Japan); LGR-pAP (Watanabe Chemical Industries, Hiroshima, Japan); 4-(2-Hydroxyethyl)-1-piperazineethanesulfonic acid (HEPES; Dojindo, Kumamoto, Japan); and negative photoresists of ZPN1150 and SU-8 3005 (Zeon, Tokyo, Japan and KAYAKU Advanced Materials, Tokyo, Japan, respectively). USP-RSE was diluted with endotoxin-free water to obtain 2 × 10^6^ EU/L of endotoxin stock solution and stored at 4 °C. Before being used, the endotoxin stock solution was vigorously mixed by vortexing for 30 min in accordance with the USP-RSE protocol. LGR-pAP was dissolved in endotoxin-free water to obtain 10 mmol/L stock solution and stored at −20 °C.

### 2.2. IDAE Device Fabrication

An IDAE device was fabricated via a lift-off process involving standard photolithography and sputtering. The photoresist of ZPN1150 was patterned on a glass slide; then, Ti/Pt/Au was sputtered on the glass slide by using a sputtering system (L-332S-FH, CANON ANELVA, Kawasaki, Japan). After lift-off in acetone, the photoresist of SU-8 3005 was patterned on the glass slide to insulate the device, except the sensor area. The IDAE device consisted of two comb-shaped electrodes as working electrodes and an Au flat plate electrode as a counter electrode on the glass slide. According to the photomask designs, the width of each comb and the distance between the combs were 10 μm, and the non-insulated length of the finger of each comb was 1.0 mm.

### 2.3. Electrochemical Measurement

Electrochemical measurements were performed using a Ag/AgCl electrode (saturated KCl) as a reference electrode and an Au flat plate as a counter electrode in a multichannel potentiostat (HA-1010 mM4, Hokuto Denko, Tokyo, Japan). A sample solution (300 μL) was introduced to the IDAE device, and the Ag/AgCl electrode was inserted into the solution. After one measurement, the IDAE device was washed with water for repeated use. Because the obtained currents from the IDAE device gradually decrease by electrode fouling with the repeated use of the device, we set the times for repeated used of an IDAE device to be up to three.

### 2.4. Endotoxin Assay

An electrochemical endotoxin assay was performed in accordance with our previously described methods [16]. The assay buffer of the Endospecy-24S set (180 μL) and 10 mmol/L LGR-pAP (20 μL) were added to a test vial containing the LAL reagents of the Endospecy-24S set to obtain the LAL solution. After the LAL solution (200 μL) and endotoxin solution (200 μL) were mixed, the LAL reaction was induced for 30 min, 45 min, or 1 h at 37 °C. Thereafter, the solution obtained after the LAL reaction (300 μL) was introduced to the IDAE device. pAP released from LGR-pAP was detected, with the potential of the anode comb stepped from −0.3 V to 0.5 V, while the cathode comb biased at −0.3 V.

## 3. Results and Discussion

### 3.1. Characterization of the IDAE Device

**Figure 2:**
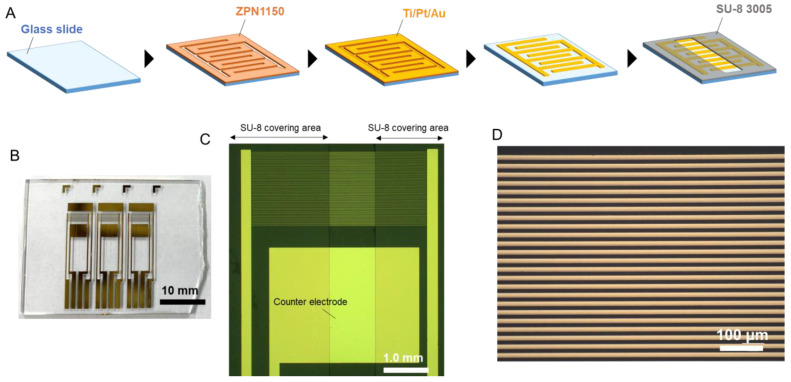
(**A**) Fabrication process for IDAE device; (**B**) Photograph of the entire IDAE device; (**C**) Optical image of IDAE device; (**D**) Magnified image of IDAE device.

**Figure 3:**
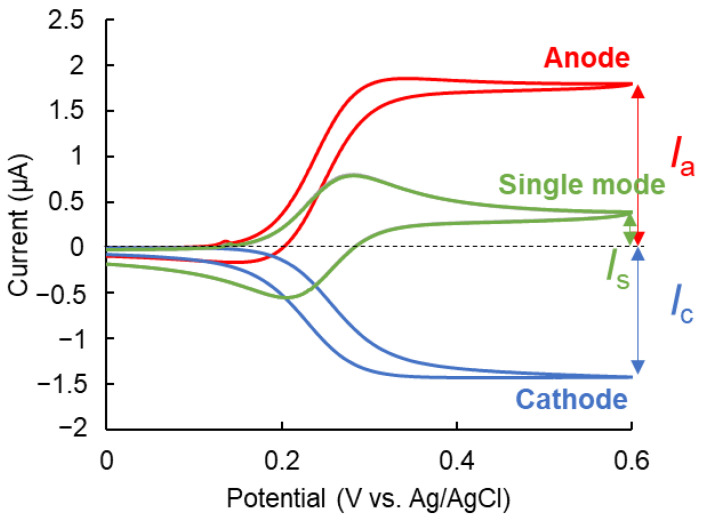
Cyclic voltammetry for 500 μmol/L ferrocenemethanol using IDAE device. Red and blue lines show voltammograms of anode and cathode with dual mode, respectively. Green line shows voltammogram of one side of comb-shaped electrode with single mode.

The IDAE device was fabricated and characterized. Figure 2A shows the fabrication of the IDAE device. Briefly, after the photoresist of ZPN1150 was patterned and Ti/Pt/Au was sputtered on the glass slide, the ZPN1150 and Ti/Pt/Au layer deposited on ZPN1150 were removed. The insulation layer of SU-8 3005 was patterned, except the sensor area. Figure 2B–D present the photograph of the whole IDAE device, as well as the optical and magnified images of the IDAE device, respectively. Three IDAE devices were constructed on a 35 mm × 27 mm glass slide. In Figure 2B, the device has two Au flat plates above and below the comb-shaped electrodes, which function as working electrodes. One Au flat plate functions as a counter electrode, while the other Au flat plate acts as a quasi-reference electrode, to achieve a portable device integrating all the electrodes on a chip device. However, a Ag/AgCl electrode was used as the reference electrode in this study instead of an Au flat plate, because the stable potential achieved by using a Ag/AgCl electrode as the reference electrode was better suited for the proof-of-concept study. In the future, further characterization of the Au flat plate as a quasi-reference electrode may result in a highly portable device. The IDAE device has a robust structure compared to the redox cycling device in a nanoscale gap [19,22,23]. The redox cycling device in a nanoscale gap can break as a result of a small impact due to the broken spatial structure, which is not the case with the IDAE device. These images reveal that the width of the comb-shaped electrode and the distance between the comb-shaped electrodes were 10 μm, and the width of the non-SU-8-covering area was 1.0 mm, which matched the photomask design. Cyclic voltammetry of 500 μmol/L ferrocenemethanol (FMA) in 0.1 mol/L KCl was performed using the IDAE device to electrochemically characterize this device. Figure 3 shows the voltammograms of FMA. The red and blue lines are the voltammograms at the anode and cathode, respectively, under the redox cycling condition called a dual mode [25,26]. The potential of the anode was swept at 50 mV/s, while the potential of the cathode was biased at 0.0 V vs. Ag/AgCl. In the voltammograms with the dual mode, the cycling efficiency (*I*_c_/*I*_a_) was 79.8%, which indicated that most of the oxidized FMA at the anode reached the cathode. The green line in Figure 3 is the voltammogram of one side of the comb-shaped electrode without the redox cycling condition, i.e., single mode [25,26]. The potential of both comb-shaped electrodes was swept at 50 mV/s. The current of both comb-shaped electrodes with the single mode was approximately equal because of the same structure. The electrochemical signal was amplified by 4.79-fold under the dual mode compared with that under the single mode (*I*_a_/*I*_s_), because of the signal amplification of redox cycling. Furthermore, the measured anodic current under the redox cycling condition was compared with the theoretical value by using Equation (1) [27]:

(1) $$I_{s}=mbnFcD\left\{0.637\ln\left(2.55w/w_{g}\right)-0.19{\left(w_{g}/w\right)}^{2}\right\}$$

In Equation (1), *I*_s_ is the steady-state current under redox cycling with the IDAE device; *m* and *b* are the number and length of the fingers of the anode comb-shaped electrode, respectively; *n* is the number of electrons involved in a redox reaction; *F* is Faraday’s constant; *c* and *D* are the concentration and diffusion coefficient of the redox molecule, respectively; and *w* and *w*_g_ are the total width of the fingers of the anode and cathode comb-shaped electrodes and the gap width between the anode and cathode, respectively. The theoretical steady-state current of the anode was 1.45 μA, where *m* = 40, *b* = 1.0 mm, *n* = 1, *F* = 9.65 × 10^4^ C/mol, *c* = 500 μmol/L, and *D* = 7.6 × 10^−10^ m^2^/s according to a previous report [28]; *w* and *w*_g_ were 20 and 10 μm, respectively, which roughly matched the steady-state current of the anode at 0.6 V vs. Ag/AgCl in Figure 3 (1.79 μA). The measured steady-state current of the anode was 0.34 μA higher than the theoretical value likely because Equation (1) does not include the effect of the height of the electrode [20]. The concentration gradients of the redox molecules near the edge of the comb-shaped electrodes had a greater height than the IDAE device, without considering the height of the electrode, which affected the signal. However, the effect of the electrode height was negligible because of the small gap between the measured and theoretical current. Therefore, the IDAE device with great redox cycling efficiency was successfully fabricated, and its structure matched the design.

### 3.2. Detection of pAP in the Presence of LGR-pAP

**Figure 4:**
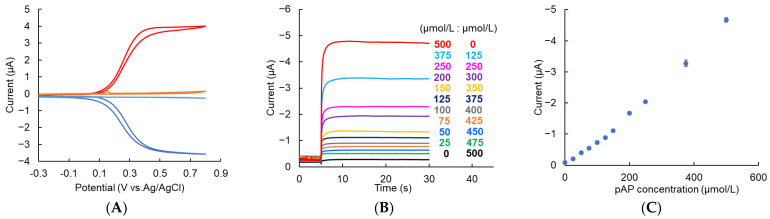
(**A**) Cyclic voltammogram for 500 μmol/L pAP and LGR-pAP using IDAE device with the potential of the anode scanned with 50 mV/s while the cathode was biased at −0.3 V vs. Ag/AgCl. Red and blue lines are the result for pAP at anode and cathode, respectively, and orange and light blue lines are the result for LGR-pAP at anode and cathode, respectively; (**B**) Amperograms obtained from cathode in mixture solution of different ratio of pAP and LGR-pAP using IDAE device. The potential of the anode was stepped from −0.3 to 0.5 V vs. Ag/AgCl at 5 s while the cathode was biased at −0.3 V vs. Ag/AgCl; (**C**) Calibration plot for pAP from current subtracting 4.96 s from 30 s in (**B**). Error bars represent standard deviation (*n* = 3).

pAP was detected in the presence of LGR-pAP to characterize our assay system because the solution after the LAL reaction contained pAP and LGR-pAP. Cyclic voltammetry for 500 μmol/L pAP or LGR-pAP in 50 mmol/L HEPES buffer (pH 7.8) containing 0.1 mol/L KCl was performed using the IDAE device to determine the applied potential for the amperometric detection of pAP in the presence of LGR-pAP. The potential of the anode was swept at 50 mV/s, while the cathode was biased at −0.3 V vs. Ag/AgCl. Figure 4A shows the results of cyclic voltammetry with the dual mode. The red and blue lines show the result of pAP at the anode and cathode, respectively. The orange and light blue lines show the result of LGR-pAP at the anode and cathode, respectively. A small peak current was observed around 0.1 V vs. Ag/AgCl, as depicted by the orange line in Figure 4A. This could be caused by the redox molecule adsorbed to the electrode surface of the IDAE device as a result of repeated use. However, this peak is negligible because of the immediate decay of the peak. These voltammograms indicated that pAP could be detected in the presence of LGR-pAP when the potentials of the anode and cathode were biased at 0.5 and −0.3 V vs. Ag/AgCl, respectively, because the potential inducing the redox cycling of LGR-pAP shifted to the positive side compared with that of pAP. pAP was amperometrically detected using a solution containing different ratios of pAP and LGR-pAP in 50 mmol/L HEPES buffer (pH 7.8) composed of 0.1 mol/L KCl as a model after the LAL reaction in the IDAE device. The potential of the anode was stepped from −0.3 V to 0.5 V vs. Ag/AgCl, while the cathode was biased at −0.3 vs. Ag/AgCl. Figure 4B shows the amperograms at the cathode. In this study, the current obtained from the cathode was characterized because cathodic current does not include noise from the charge of the electric double layer. Because the cathode was biased at a constant potential, the cathodic current indicates only the faradic current. After the potential step at the anode, a steady-state current was obtained in a few seconds because a diffusion layer between the anode and cathode was formed in a short time after the potential step. Figure 4C shows the calibration plots of pAP from the current subtracted at 4.96 s as a background signal by reducing oxygen from 30 s in Figure 4B. The amplified current by the redox cycling of pAP increased as the pAP ratio increased. Thus, pAP was successfully detected quantitatively in the presence of LGR-pAP by using the IDAE device when the potential of the anode was stepped from −0.3 V to 0.5 V vs. Ag/AgCl and that of the cathode was biased at −0.3 V vs. Ag/AgCl.

### 3.3. Endotoxin Assay

**Figure 5:**
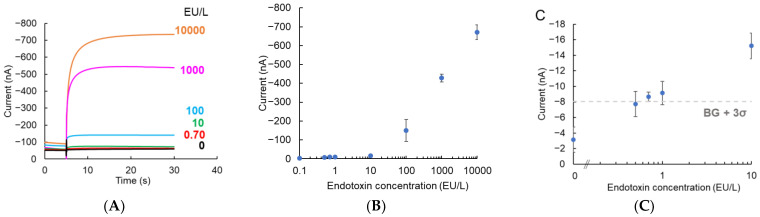

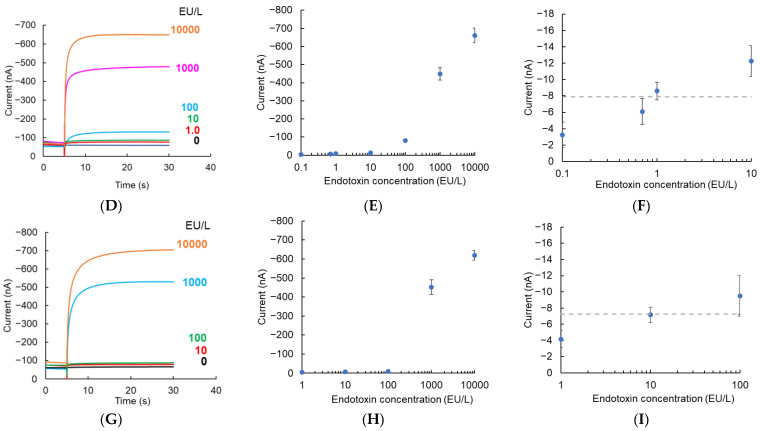
(**A**) Amperograms obtained from cathode with 1 h of LAL reaction time; (**B**,**C**) Calibration plot for endotoxin with 1 h of LAL reaction time and magnified calibration plot, respectively; (**D**–**F**) Similarly, with a reaction time of 45 min and (**G**–**I**) 30 min. The potential of the anode was stepped from −0.3 to 0.5 V vs. Ag/AgCl at 5 s while the cathode was biased at −0.3 V vs. Ag/AgCl. The plotted values are current subtracted at 4.96 s from 30 s of amperograms. BG + 3σ indicates three times of standard deviation of 0 EU/L of endotoxin. Error bars represent standard deviation (*n* = 3).

The endotoxin assay was performed using the IDAE device. The LAL reaction with the endotoxin was induced using 0.5 mmol/L LGR-pAP for 1 h, 45 min, or 30 min at 37 °C. Thereafter, pAP released from LGR-pAP was detected using the IDAE device when the potential of the anode was stepped from −0.3 V to 0.5 V vs. Ag/AgCl, while the cathode was biased at −0.3 V vs. Ag/AgCl. Figure 5A shows the amperograms of the endotoxin obtained from the cathode with 1 h of the LAL reaction. Figure 5B presents the calibration plots of the endotoxin with 1 h of the LAL reaction time from the current subtracted at 4.96 s from 30 s in Figure 5A. The amplified current by the redox cycling of pAP increased as the endotoxin concentration increased. Figure 5C shows the magnified calibration plots from Figure 5B. BG + 3σ is three times the standard deviation of 0 EU/L of endotoxin. Based on the BG + 3σ line, the limit of detection of endotoxin with 1 h of the LAL reaction was 0.7 EU/L, which improved by 14-fold compared with that obtained in our previous study using an electrochemical chip device with a carbon disk electrode [16]. This sensitivity improvement was attributed to signal amplification via redox cycling by using the IDAE device. Figure 5D shows the amperograms of the endotoxin with 45 min of the LAL reaction. Figure 5E,F present the calibration plots and magnified calibration plots of endotoxin with 45 min of the LAL reaction, respectively. The plotted value was determined using the current subtracted at 4.96 s from 30 s in Figure 5D. The limit of detection of endotoxin with 45 min of the LAL reaction time was 1.0 EU/L based on the BG + 3σ line. Therefore, our endotoxin sensor using the IDAE device had a limit of detection covering the cut-off value of ultrapure dialysis fluid (1.0 EU/L) with 1 h and 45 min of the LAL reaction. Figure 5G shows the amperograms of endotoxin with 30 min of the LAL reaction. Figure 5H and I show the calibration plots and magnified calibration plots of endotoxin with 30 min of the LAL reaction, respectively. Based on the BG + 3σ line, the limit of detection of endotoxin with 30 min of the LAL reaction was 100 EU/L, which was insufficient for 1.0 EU/L because the amount of pAP released from LGR-pAP with 30 min of the LAL reaction was inadequate for detection via redox cycling using the IDAE device. In addition, the endotoxin sensor with the device for redox cycling in a nanoscale gap detected values as low as 0.5 EU/L with 30 min of the LAL reaction time in our previous study [19], which showed that the sensitivity of the sensor in this study was less than the endotoxin sensor using a device with a nanoscale gap in our previous study. The redox cycling efficiency increases as the electrode distance decreases [29]. The electrode distance of 10 μm of the IDAE device in this study was larger than the 190 nm of the device for redox cycling in a nanoscale gap, which negatively affected sensitivity. However, the IDAE device has the advantages of robustness and the clean shape of the amperograms. Additionally, an IDAE device with a 10 μm electrode distance is easier to produce than a device for redox cycling in a nanoscale gap because devices with a small electrode distance are difficult to manufacture, which is an important factor when considering mass production. In our previous study involving a device for redox cycling in a nanoscale gap [19,22,23], the shape of amperograms was influenced by the adsorption and desorption of a redox molecule to the electrode surface [30]. A LAL reagent without a chromogenic substrate can be effectively used to obtain 1.0 EU/L of the limit of detection with 30 min of the LAL reaction by using the IDAE device. In this study, a commercial chromogenic LAL assay kit containing LGR-pNA as a chromogenic substrate in the LAL reagent was used. Therefore, the LGR-pAP reaction catalyzed by a clotting enzyme in the last step of the LAL reaction was competed with LGR-pNA, which limited the sensitivity of the endotoxin sensor. The sensitivity of the endotoxin sensor under the LAL reaction for 1 h in this study was compared with that of previously reported endotoxin sensors (Table 1). Our endotoxin sensor using the IDAE device was more sensitive than most of the endotoxin sensors. The linear range of detection is an important parameter for the analytical performance of sensors. However, the relationship between endotoxin concentration and signals is not linear in LAL-based endotoxin sensors because of the nature of the cascade reaction [16,18,19]. Therefore, our sensor is required to perform curve fitting on the calibration plots. For further characterization of performance of our endotoxin sensor, in the future we will compare the endotoxin assay results obtained using the IDAE device with those from the conventional LAL-based endotoxin assay. Our endotoxin sensor using the IDAE device is considered to have a high selectivity towards endotoxins, because of the high selectivity of the LAL reaction towards endotoxins, and the property of inducing redox cycling with only reversible redox species. In the future, we will characterize the selectivity of our sensor using various interferences. Additionally, in the future we will characterize the application ability of our endotoxin sensor in real samples, such as dialysis water. Our sensor will successfully perform endotoxin assays using dialysis water because, according to United States Pharmacopeia [31], calibration plots for endotoxins obtained using endotoxin standard solutions prepared with endotoxin-free water are used for endotoxin tests in dialysis water. Therefore, a highly sensitive endotoxin sensor was successfully developed via redox cycling by using the IDAE device.

## 4. Conclusions

In this study, a highly sensitive endotoxin sensor involving redox cycling was developed by using an IDAE device. The characterization of the fabricated IDAE device indicated that the IDAE device had a great redox cycling efficiency of 79.8% and 4.79-fold signal amplification rate. Cyclic voltammetry and chronoamperometry for pAP and LGR-pAP in HEPES buffer revealed that pAP was detectable in the presence of LGR-pAP, and the potential of the anode and cathode could be biased at 0.5 and −0.3 V vs. Ag/AgCl, respectively. The endotoxin assay demonstrated that values as low as 0.7, 1.0, and 100 EU/L endotoxin could be detected after the LAL reaction of 1 h, 45 min, and 30 min, respectively. The limit of detection of our sensor covered the cut-off value of ultrapure dialysis fluid (1.0 EU/L) with 1 h and 45 min of the LAL reaction time. In the future, the sensitivity of our sensor will be improved to reach 1.0 EU/L with 30 min of the LAL reaction time by using a LAL reagent without a chromogenic substrate and an IDEA device with reduced electrode distance for improved redox cycling efficiency. In the future, we will perform real sample analysis, selectivity characterization, and a comparison of analytical performance of our sensor with the conventional LAL-based endotoxin assay for the realization of practical use. Our highly sensitive endotoxin sensor will be used to ensure medical safety.

## Figures and Tables

**Table 1:** Comparison of limit of detection of endotoxin sensor.

**Detection Method**	**Limit of Detection**	**Reference**
Differential pulse voltammetry	0.2 EU/L	[32]
Impedance spectroscopy	1.0 EU/L	[33]
Amperometry	70 EU/L	[34]
Square wave voltammetry	10 EU/L	[35]
Differential pulse voltammetry	40 EU/L	[36]
Impedance spectroscopy	30 EU/L	[37]
Magnetoelastic sensor	10.5 EU/L	[38]
Amperometry	0.5 EU/L	[19]
Amperometry	0.7 EU/L	This study

**Detection Method**

**Limit of Detection**

**Reference**

## Data Availability

The data presented in this study are available upon request from the corresponding authors.
